# Appropriateness of Percutaneous Coronary Interventions: A Systematic Review and Meta-Analysis

**DOI:** 10.3390/jcdd10030093

**Published:** 2023-02-21

**Authors:** Yijie Liu, Yuxiong Chen, Zhen’ge Chang, Yitao Han, Siqi Tang, Yakun Zhao, Jia Fu, Yanbo Liu, Zhongjie Fan

**Affiliations:** 1 Department of Cardiology, Peking Union Medical College Hospital, Peking Union Medical College and Chinese Academy of Medical Sciences, Beijing 100730, China; 2 Department of Respiratory, Civil Aviation General Hospital, Beijing 100123, China

**Keywords:** appropriate use criteria, percutaneous coronary interventions, patient selection, appropriateness, coronary artery disease, coronary revascularization

## Abstract

Background: Since the foundation of appropriate use criteria (AUC) for coronary revascularization, the proportion of inappropriate (later revised as “rarely inappropriate”) percutaneous coronary interventions (PCIs) varied in different populations. However, the pooled inappropriate PCI rate remains unknown. Methods: We searched the PubMed, Cochrane, Embase, and Sinomed databases for studies related to AUC and PCIs. Studies that reported inappropriate/rarely appropriate PCI rates were included. A random effects model was employed in the meta-analysis because of the high statistical heterogeneity. Results: Thirty-seven studies were included in our study, of which eight studies reported the appropriateness of acute PCIs or PCIs in acute coronary syndrome (ACS) patients, 25 studies reported the appropriateness of non-acute/elective PCIs or PCIs in non-ACS/stable ischemic heart disease (SIHD) patients, and 15 studies reported both acute and non-acute PCIs or did not distinguish the urgency of PCI. The pooled inappropriate PCI rate was 4.3% (95% CI: 2.6–6.4%) in acute scenarios, 8.9% (95% CI: 6.7–11.0%) in non-acute scenarios, and 6.1% (95% CI: 4.9–7.3%) overall. The inappropriate/rarely appropriate PCI rate was significantly higher in non-acute than acute scenarios. No difference in the inappropriate PCI rate was detected based on the study location, the country’s level of development, or the presence of chronic total occlusion (CTO). Conclusions: The worldwide inappropriate PCI rate is generally identical but comparatively high, especially under non-acute scenarios.

## 1. Introduction

Cardiovascular diseases (CVDs) remain the leading cause of death globally [1]. Coronary artery disease (CAD) has been found to be the leading cause of death in both developed and developing countries [2]. In the US, CAD is the most common type of heart disease, killing 382,820 people in 2020 [3]. In China, the estimated number of CAD patients was 11.39 million people in 2018, with a mortality rate of 120.18/100,000 [4]. Percutaneous coronary intervention (PCI) has evolved dramatically and is continuously an acceptable treatment option for patients with advanced CAD [5]. The increasing prevalence of CAD, advances in surgical and percutaneous techniques for revascularization, concomitant medical therapy for CAD, and the costs of revascularization have resulted in heightened interest regarding the appropriateness of coronary revascularization. The hazard of overuse of healthcare services outweighs the benefits [6], especially for invasive operations. Appropriateness criteria are designed to examine the use of diagnostic and therapeutic procedures to support the efficient use of medical resources during the pursuit of quality medical care [7]. Over the past two decades, many countries have developed appropriate use criteria (AUC) for coronary revascularization according to the local patients’ characteristics and PCI guidance, which are updated regularly to address the expanding clinical indications for coronary revascularization [8].

A series of studies has examined the appropriateness of PCI, nevertheless, the inappropriate/rarely appropriate PCI rate on the whole remains unknown. A systematic review and meta-analysis is required to summarize the inappropriate/rarely appropriate PCI rate for further AUC updates; to encourage more local AUC; and to instruct, standardize, and supervise the use of PCIs.

## 2. Materials and Methods

### 2.1. Data Source and Search Strategy

We searched the following sources from inception to July 2022. Studies were identified from the following electronic databases without a language restriction: PubMed, Cochrane, and Embase. Sinomed was searched using Chinese only. We also searched the reference lists of identified studies for relevant articles. The terms “myocardial revascularization”, “percutaneous coronary intervention”, “patient selection”, “coronary artery disease”, and “appropriate use criteria” were included. MeSH, Emtree, and other theme words were used as a major search strategy in the corresponding databases. The search strategies are provided in Item S1 in the Supplementary Materials. Our study was previously registered at PROSPERO with the register ID: CRD42022348359.

### 2.2. Appropriate Use Criteria for PCI

All versions of published AUC were included (America 2009 AUC [7], America 2012 AUC [7], America 2017 AUC [8], China 2016 AUC [9], Republic of Korea 2017 AUC [KP3] [10], and Japan 2007 AUC [11]). Local AUCs were preferred due to an improved ability to map patients to the AUC. The rating process and scoring of the AUC by panelists were generally based on the RAND method (a modified Delphi process) [12], using the following definition of appropriate use [13]:

**Definition** **1.** *Coronary revascularization is appropriate when the expected benefits, in terms of survival or health outcomes (symptoms, functional status, and/or quality of life), exceed the expected negative consequences of the procedure*.

### 2.3. Inclusion and Exclusion Criteria

To meet the analysis requirements and to reduce deviation, selected studies fulfilled the following criteria: (1) the study was based on population samples rather than volunteers, and enough information could be acquired from the article or the author; (2) the AUC were declared or could be inferred from the contents; (3) if there were multiple articles based on the same sample, the inclusion criteria were described in the following sequence: ① when the same sample was mapped to local and abroad AUC simultaneously or respectively, the cohort using the local AUC was included; ② when more than one study was based on exactly the same sample, the one using the most comprehensive data was included; ③ when different studies from different cohorts might have contained the same sample, all studies were included.

Major exclusion criteria: review or case report; reporting the appropriateness of revascularization for procedures other than PCI; reporting modified AUC; and reporting AUC scores only. In addition, studies excluded due to duplications in non-acute PCI/SIHD patient group could be included in the acute PCI/ACS patient group, or vice versa. We contacted the authors of primary studies if the original articles failed to contain enough information to enable an accurate assessment of eligibility for inclusion. If no reply regarding the data requirements was received within 30 days, the article was also excluded from our study.

### 2.4. Quality of the Studies and Risk of Bias Assessment

Two review authors (Yijie Liu and Y. Chen) independently evaluated the quality of included studies using the Methods Guide for Comparative Effectiveness Reviews of the US Agency for Healthcare Research and Quality (AHRQ) [14]. The answer “yes” scored 1 point while “unclear” or “no” did not score any points. Eleven domains of bias were assessed. Based on the reviewers’ judgments, every article was rated as having a high (0–3 points), medium (4–7 points), or low risk (8–11 points) classification.

### 2.5. Data Analysis

We used published systematic analysis techniques to calculate the pooled inappropriate (also described as “rarely appropriate” in later studies) acute PCI rate and the non-acute PCI rate. Due to the high statistical heterogeneity in single-group meta-analyses, a random effects meta-analysis was performed in all groups to increase the robustness. To minimize the heterogeneity produced by the absence of a control group in the single-group meta-analyses, subgroup analyses were performed based on location (Asia vs. North America), country’s level of development (developing vs. developed countries), and specific type of CAD present (CTO [chronic total occlusion] patients vs. other SIHD patients). To increase robustness, the meta-analysis of single rates with zero events was based on the Freeman-Tukey transformation [15]. Publication bias was examined by the Egger’s test. Risk of bias assessments were counted using Review Manager 5.4 (The Cochrane Collaboration, 2020). All meta-analyses were performed using Stata Statistical Software 12 (StataCorp.; College Station, TX, USA).

## 3. Results

### 3.1. Characteristics of Included Studies

**Figure 1:**
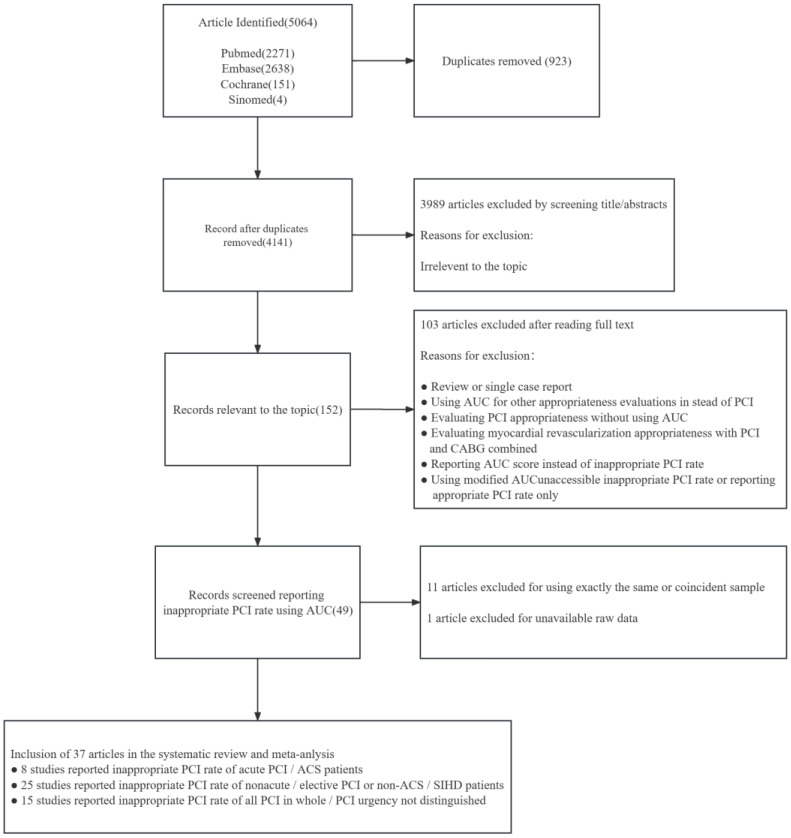
Flow chart of the meta-analysis exclusion/inclusion criteria for individual articles.

The process of our search strategy is illustrated in Figure 1. Thirty-seven studies were included in our review. Eight studies reported the appropriateness of acute PCIs or ACS patients, 25 studies reported the appropriateness of non-acute PCIs/elective PCIs or non-ACS/SIHD patients (four studies reported the appropriateness of PCIs in CTO patients), and 15 studies reported the appropriateness of both acute and non-acute PCIs or did not distinguish the urgency of PCI. Among 37 included studies, 22 were performed in North America (19 in the US, two in Canada, and one in both Canada and the US), 11 were performed in Asia (four in Japan, three in India, one in the Republic of Korea, one in Pakistan, one in China, and one in Indonesia), and four in other regions (one in Brazil, one in the UK, one in Russia, and one in Italy). Eight studies were performed in developing countries.

Table 1 shows the characteristics of the included studies. The risk of bias graph and the risk of bias summary are shown in the appendix (Supplementary Figures S1 and S2, respectively). In most studies, inappropriate/rarely appropriate non-acute PCI rates were significantly higher than acute PCI rates [16,17,18,19,20,21] (shown in Table 2). In our study, the pooled inappropriate PCI rate in acute scenarios was significantly lower than in non-acute scenarios (*p* = 0.023).

### 3.2. Meta-Analysis Results

#### 3.2.1. Inappropriate PCI Rate of Acute PCI/ACS Patients

**Figure 2:**
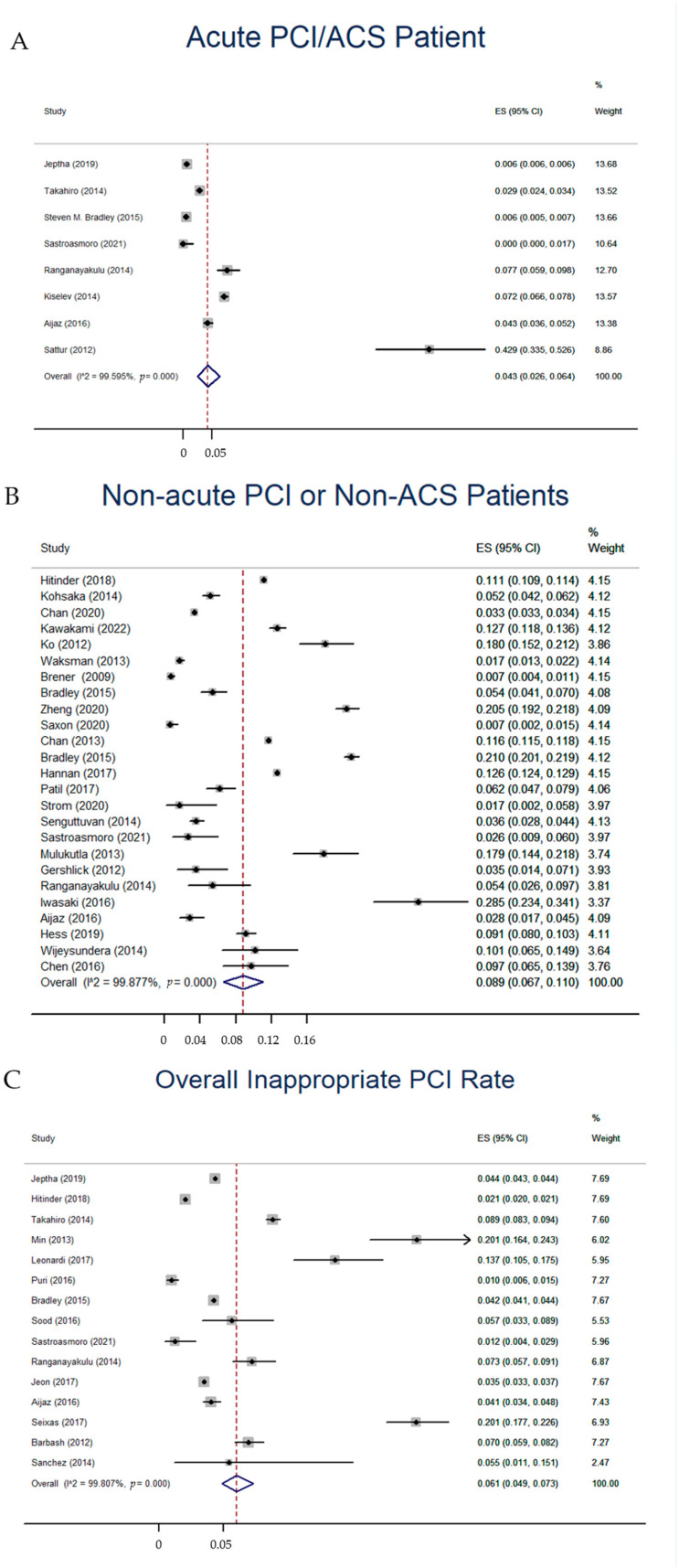
Forest plot for the meta-analysis of the inappropriate rate of PCI. (**A**) Pooled inappropriate PCI rate of acute PCI/ACS patients. (**B**) Pooled inappropriate PCI rate of non-acute/elective PCI or non-ACS/SIHD patients. (**C**) Pooled inappropriate PCI rate among patients in whole/PCI urgency not distinguished.

A total of eight studies with 990,910 acute PCIs were included in our study [17,18,19,21,22,35,43,45]. The America 2012 AUC were used in six studies while the America 2009 AUC [45] and America 2017 AUC [21] were each used in one study, respectively. Three studies were performed in the US [17,18,45], one in Russia [35], one in Japan [19], one in Pakistan [22], one in India [43], and one in Indonesia [21]. The pooled inappropriate PCI rate was 4.3% (95%CI: 2.6–6.4%) among acute PCI or ACS patients (Figure 2A). A Freeman-Tukey transformation was used in the case of zero events. Publication bias was significant (*p* = 0.018) in acute PCI or ACS patients. Further trim and filling showed that the pooled inappropriate PCI rate was not significantly affected by the publication bias.

#### 3.2.2. Inappropriate PCI Rate of Non-Acute/Elective PCI or Non-ACS/SIHD Patients

A total of 25 studies with 597,843 non-acute/elective PCIs were included. The America 2012 AUC were used in 11 studies [18,22,24,28,30,31,32,33,41,43,46], the America 2009 AUC were used in nine studies [25,26,27,29,36,40,48,51,52], the America 2017 AUC were used in three studies [21,34,50], and the China 2016 AUC and Japan 2007 AUC were both used once [37,53]. Thirteen studies were performed in the US [18,25,26,27,28,30,31,32,40,46,48,50,51], three in Japan [33,34,37], two in India [41,43], two in Canada [36,52], one in China [53], one in Pakistan [22], one in Indonesia [21], one in the UK [29], and one in both Canada and the US [24]. Five studies were performed in developing countries [21,22,41,43,53]. The pooled inappropriate non-acute PCI rate was 8.9% (95% CI: 6.7–11.0%) in the included studies (Figure 2B). Publication bias was not significant (*p* = 0.322, funnel plot in Figure S3) in non-acute/elective PCIs.

#### 3.2.3. Inappropriate PCI Rate of All PCIs/PCI Urgency Not Distinguished

Fifteen studies reported the inappropriate PCI rate in both acute and non-acute scenarios or did not distinguish the urgency of PCI. A total of 1,721,811 PCIs were included. Seven studies were performed in the US [17,18,23,30,39,42,44], one in the Republic of Korea [10], two in India [43,49], one in Japan [19], one in Pakistan [22], one in Brazil [47], one in Italy [38], and one in Indonesia [21]. Five studies were performed in developing countries. The pooled inappropriate overall PCI rate was 6.1% (95% CI: 4.9–7.3%) (Figure 2C). Publication bias was not significant (*p* = 0.512, funnel plot in Figure S4).

### 3.3. Sub-Group Analyses

**Figure 3:**
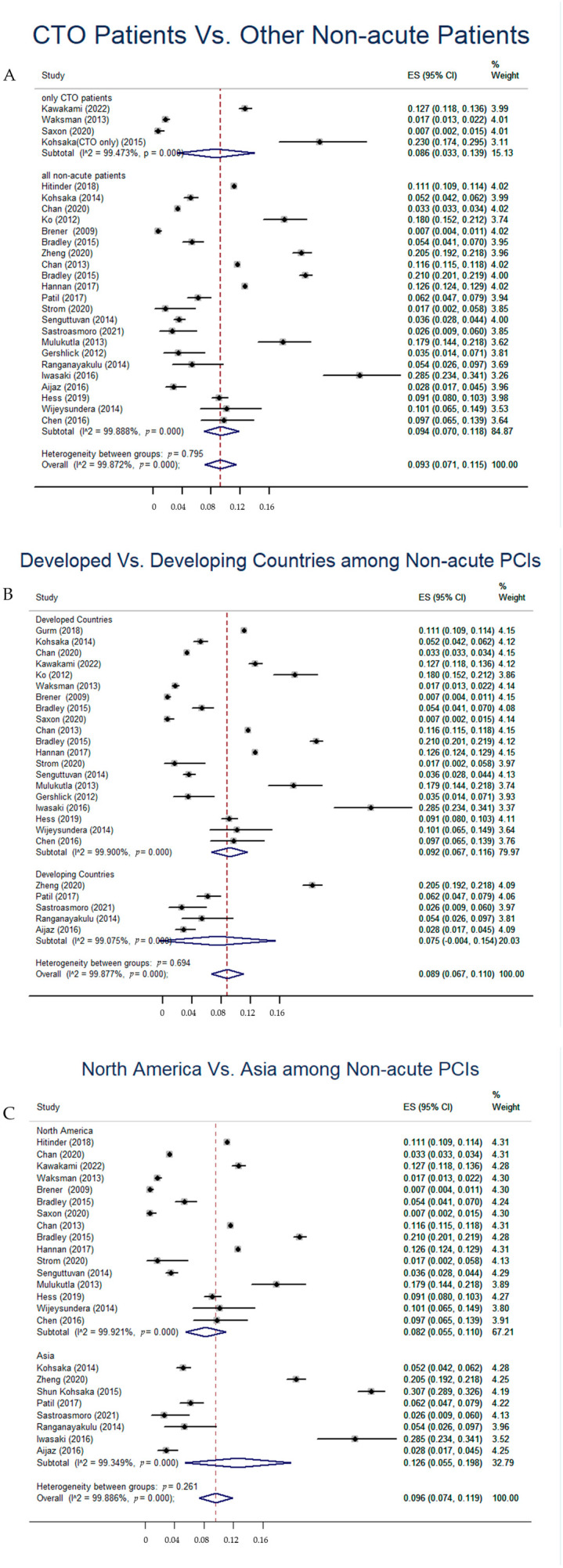
Subgroup analyses for the heterogeneity of included studies. (**A**) Inappropriate PCI rate between CTO patients and other non-acute PCI patients. (**B**) Inappropriate non-acute PCI rate in developed countries and developing countries. (**C**) Inappropriate non-acute PCI rate in North America and Asia.

Sub-group analyses were performed to examine the heterogeneity due to study location, the country’s degree of development, and the presence of CTO. The result of the analysis involving CTO patients versus other non-acute PCI patients is shown in Figure 3. The pooled inappropriate PCI rate was 8.6% (95% CI: 3.3–13.9%) in CTO patients while it was 9.4% (95% CI: 7.0–11.8%) in other non-acute PCI or non-ACS patients. No significant difference was found between the two groups (*p* = 0.795, Figure 3A). The inappropriate non-acute PCI rate in developing countries was 7.5% (95% CI: 0–15.4%) while it was 9.2% (95% CI: 6.7–11.6%) in developed countries. No significant difference was found between the two groups (*p* = 0.694, Figure 3B). Given that most research was performed in Asia and North America, we compared the inappropriate PCI rate between these two groups. Among non-acute PCIs, the overall inappropriate PCI rate was 8.2% (95% CI: 5.5–11.0) in North America while the overall inappropriate PCI rate was 12.6% (95% CI: 5.5–19.8%) in Asia. No significant difference was found between the two groups (*p* = 0.261, Figure 3C). The meta-regression results corresponded with the sub-group analyses (*p* > 0.05, respectively).

## 4. Discussion

In this meta-analysis of 37 studies, including around 2 million patients, we reported the inappropriate PCI rate in acute and non-acute circumstances, respectively. The pooled inappropriate PCI rate was 4.3% among acute PCI or ACS patients and 8.9% among non-acute PCI or SIHD/non-ACS patients. The pooled inappropriate PCI rate in acute scenarios was significantly lower than in non-acute scenarios (*p* = 0.023). Most of the non-acute procedures classified as inappropriate were performed in settings where the benefit of PCI has not been demonstrated and in most cases, they happened in non-acute scenarios [16]. In addition, the overuse of coronary revascularization was more likely to occur in non-acute scenarios.

Sub-group analyses were performed in non-acute PCI or non-ACS patients and found no significant difference in the inappropriate PCI rate when the studies were stratified by the country’s level of development, the presence of CTO, or the region of study. Hospital level, insurance status, demographic characteristics of the patients, and AUC type were reported as interfering factors of the inappropriate PCI rate.

### 4.1. Interfering Factors of the Inappropriate PCI Rate

#### 4.1.1. The Factor of Hospital Level

Within the same country, higher ranked hospitals had higher inappropriate PCI rates than lower ranked ones. Chan et al. [16] reported that under non-acute conditions, hospitals in the lowest quartile had inappropriate PCI rates of 6% or lower, while the rates were greater than 16% among hospitals in the highest quartile (median RR: 1.80). Patients admitted at rural hospitals were less likely to undergo an inappropriate PCI than those at urban hospitals (adjusted OR: 0.92) [26]. Cardiologists in low-volume PCI centers are likely to strictly examine the indication and appropriateness of PCI before making a conclusive clinical decision, resulting in a lower inappropriate PCI rate. More importantly, patients with non-acute PCI demands might spontaneously visit high-volume, experienced PCI centers for higher quality PCIs, which could result in sampling bias. Although the medical conditions varied, we found no difference in the inappropriate non-acute PCI rate between developing and developed countries. It was also reassuring to see that hospital-level appropriateness was not related to clinical outcomes [30,54]. For acute scenarios, a short door-to-balloon time (DBT, ≤90 min) was associated with a lower mortality rate in patients with an early presentation [55]. AUC could therefore be an effective tool for promoting the extension of local low-volume PCI centers, avoiding prolongation of the DBT, and relieving the pressure in high-volume PCI centers. Qualitative studies of hospitals with higher inappropriate PCI rates may also serve to validate the importance of the patient selection processes identified at hospitals with better PCI appropriateness rates [18].

#### 4.1.2. The Factor of Insurance Status

The implementation of PCIs could be affected by insurance status [26]. Chan et al. [26] found that for non-acute indications, patients in the US without insurance (adjusted OR: 0.56) were the least likely to undergo an inappropriate PCI (*p* < 0.001). Lack of health insurance was reported to be associated with delays in seeking emergency care for AMI [56], and the absence of private insurance potentially indicated that the patients needed to bear heavy financial burdens related to PCIs. In addition, insurance programs, especially those provided by the government for the commonfolk, were likely to have stricter reimbursement thresholds and supervision systems. Surgeons and hospitals might have managed to lower the inappropriate PCI rate so that fewer claims were rejected. A Medicare PCI cohort reported comparable increases in coding for AMI and corresponding decreases in coding for SIHD and non-ACS indications [57]. These findings led to the concern that AUC may have incentivized some cardiologists to upcode stable angina to UA to conform to the AUC. Such practices damaged the credibility of the profession, increased healthcare spending, violated patient autonomy, put patients at risk of procedural complications, and may have even crossed the threshold into criminal activity [58]. Further studies are needed to fully understand the relationship between PCIs and health insurance so that the healthcare security administrations and insurance companies could apply AUC more properly to the reimbursement policies.

#### 4.1.3. The Factor of Patients’ Characteristics

Chan et al. [26] reported that for non-acute indications, men (adjusted OR: 1.08) and whites (adjusted OR: 1.09) were more likely to undergo an inappropriate PCI in the US, which corresponded to a previous study [59]. However, racial and sex differences in PCI rates may not be solely due to underuse, but also overuse. Insurance status and financial conditions were potential confounding factors in the analysis of the patients’ characteristics. In addition, complications may influence the appropriateness of PCI. Patients with heart failure, left ventricular dysfunction, or known CAD were less likely to undergo an inappropriate PCI, whereas patients undergoing a pre-operative evaluation for non-cardiac surgery were more likely to undergo a PCI categorized as inappropriate [26]. Furthermore, probably due to the effort to reduce contrast administration, ACS patients with CKD received optimal medical treatment and early invasive strategies less frequently than did other patients [60]. The risk affordability (e.g., contrast-induced nephropathy) of the patients and physicians may count in these cases. In cases with multiple or complex complications, the final implementation of PCI tended to go through a more thorough evaluation or was part of MDT (multi-disciplinary treatment), resulting in a lower inappropriate PCI rate. Some procedures classified as appropriate may be inappropriate in a particular clinical situation, such as a patient with a limited life expectancy or end-stage renal disease [16], which made the pooled inappropriate PCI rate a more complicated dependent variable.

#### 4.1.4. The Factor of Specific Type of CAD

PCIs for lesions with CTO were believed to perform better under more rigorous indications than those without CTO, which could contribute to lower inappropriate PCI rates. However, our study found no difference in the pooled inappropriate PCI rate between CTO patients and general non-ACS patients in the meta-analysis. The majority of CTO patients were not distinguished from overall non-ACS patients in most studies. Kohsaka et al. [61] reported that CTO PCIs were performed for fewer inappropriate indications than PCIs for lesions without CTO (*p* = 0.04). Saxon et al. [46] and Waksman et al. [51] reported significantly lower inappropriate PCI rates in CTO patients. The AUC methodology could be a reasonable framework for clinical decision-making when considering PCI of CTO patients.

### 4.2. Inappropriate PCI and Patients’ Outcomes

In current research, inappropriate PCI generally had no influence on patients’ outcomes, suggesting that the PCI appropriateness measures were independent of how well the procedure was performed [54]. A hospital’s proportion of inappropriate PCIs was neither associated with in-hospital mortality, bleeding, or medical therapy at discharge [54]; nor with long-term outcomes [51]; including major adverse cardiovascular events (MACE), death, MI, and target vessel revascularization (TVR) [48]. even with a prolonged, up to 900-day-long, observation period [25]. Hospital-level AUC scores did not correlate with 90-day readmission, mortality, or episode costs [62]. Interestingly, stable CAD patients with unclassified appropriateness scores showed improved outcomes with revascularization [63].

Studies outside of the US reported similar results regarding patients’ outcomes. A study in China [53] found no benefit of coronary revascularization in patients with inappropriate indications compared with a medical therapy group according to the Chinese AUC. Studies in Japan [34] showed that the rarely appropriate CTO PCIs were not associated with the incidence of major adverse cardiac and cerebrovascular events (MACCE). There was no difference in the occurrence of target lesion revascularization (TLR) in an inappropriate PCI group six months after PCI [33]. In Brazil, no difference in procedural complications and 2-year MACE among three appropriateness groups was found [47].

The AUC classification list is a valuable tool within the quality assurance process. It is vital that interventionists ensure that PCI case selection is both evidence-based and patient-oriented [64]. It was astonishing to see that the inappropriate PCI rate failed to predict the outcome in CAD patients in our study. Potential reasons for this include:

(1) Insufficient following time. The COURAGE Trial [65] did not find a difference in survival between an initial PCI plus medical therapy vs. medical therapy alone in patients with SIHD during an extended follow-up period of 15 years. However, the current study generally reported the incidence of MACCE or death after 1 to 3 years of follow-up. A prolonged follow-up time is needed in the future to observe a smaller difference in the outcomes associated with appropriate vs. inappropriate PCIs.
(2) The limitation for inappropriate PCI in predicting patients’ outcomes. The AUC classification list is only a tool to assist in clinical decision-making and should not be the sole determinant of patient care [46]. In addition to clinical indications, the quick relief of symptoms by PCI and the patient’s selection make PCI a joint, shared, and individual decision. The current AUC failed to contain all interfering factors. Among general assumptions in the America 2012 AUC, no unusual extenuating circumstances, such as an inability to comply with antiplatelet agents or a patient’s unwillingness to consider revascularization, existed; however, these bothering scenarios are often part of routine cases. Appropriate PCIs were reported with positive effects on the patients’ outcomes [53]. Further studies are required to examine the correlation between inappropriate/appropriate PCIs and patients’ outcomes among specific populations, such as patients with heart failure, CKD, etc.
(3) The effect of AUC and symptom relief. Angina relief, quality of life, and other “soft endpoints”, which are difficult to quantify but are of deep concern to patients [64], may count in future studies due to their lack of association with AUC and “hard endpoints”. Multiple studies reported that patients in the appropriate group had greater improvements in Seattle Angina Questionnaire (SAQ) [66] scores at 1 year [24,46]. A prolonged follow-up time is needed in future studies using SAQ and other symptom-related evaluations. Hard and soft endpoints should be evaluated simultaneously in every cohort to guarantee the effectiveness and reliability of the soft endpoints.

### 4.3. AUC in Real-World Clinical Practice

The appraisal of PCI use is warranted on account of its universality, expenditure, and in some cases, scarcity. A patient’s clinical presentation, including their clinical acuity, symptom severity, adequacy of antianginal therapy, ischemic risk by non-invasive testing, and severity of anatomic coronary disease, jointly determine the appropriateness of PCI [7,13]. Due to the reliance of AUC on specific clinical characteristics, physicians could use ischemic symptoms (classified by the CCS [Canadian Cardiovascular Society, Ottawa, ON, Canada]), anti-ischemic medical therapy status, non-invasive test results status, prior coronary artery bypass grafting (CABG) status, or TIMI ACS risk score to calculate the AUC score using an existing algorithm [67] for a quick appropriateness check. The fast approach to determine the appropriateness of PCI offers a more suitable application of AUC and can provide both an assessment of care decisions in aggregated patient populations and feedback to providers regarding how their individual care decisions match those from a larger population [68].

AUCs are intended to assist patients and clinicians but are not intended to diminish the acknowledged difficulty or uncertainty of clinical decision-making and cannot act as substitutes for sound clinical judgment and practical experience [13]. The current AUC should be viewed as an evaluation of the evidence base and the rational use of cardiovascular technologies in patient populations. The focus is to encourage optimal patient care via professional stewardship of technology utilization within cardiovascular medicine. Services rated as “appropriate” should be considered reasonable but not necessarily required. Services rated as “may be appropriate” should be performed depending on the clinical circumstances of the patient as well as the patient and provider preferences and should include shared decision-making due to a limited quality or quantity of evidence for specific patients. Services classified as “rarely appropriate” (previously rated as “inappropriate”) reflect the complexity of patient care. Procedures in this category should be justified using the patient’s unique circumstances, which should be documented adequately [68].

One of the major differences between AUC in America and Asia is scenario identification for patients without stress tests, which is commonly used for both diagnosis and risk stratification of patients with CAD in the US, but is rarely used in Asia [69]. This means that patients receiving PCIs without prior stress tests are unmappable to the American AUC. The Chinese 2016 AUC [9] classify stress testing status as “no stress testing”, “stress testing negative”, or “stress testing positive” so that patients without stress tests are mappable to the AUC [69]. Results from Asia [19] revealed that CT-based procedures could hypothetically be graded as appropriate instead of inappropriate. Further studies are needed to evaluate the appropriateness of CT-guided PCIs, which may suggest that a revision of the AUC is reasonable.

#### 4.3.1. AUC and Cost Savings

Over three quarters of CVD-related deaths take place in low- and middle-income countries [1], which makes it crucial to take the cost of PCI seriously. Hospital-level AUC scores did not correlate with episode costs [62] while AUC did play a role in cost savings. Puri et al. [42] found that after the implementation of the AUC in 2012, the total hospital reimbursement for coronary interventions decreased by 26% from 2011 to 2012 and decreased by a further 14% in 2013, leading to cost savings of more than $2.3 billion to the Medicare system [70]. Given that existing studies did not find a significant relationship between patients’ outcomes and AUC, insurance companies and medical insurance offices using AUC adherence to adjust reimbursement rates may be considered as engaging in unreasonable and misleading practices. Before the publication of more conclusive evidence, insurance companies should not reject the claims of patients with indications to undergo inappropriate/rarely appropriate PCIs for clinical benefit and potential symptom improvement.

#### 4.3.2. Changes in the PCI Rate and Inappropriate PCI Rate after the Implementation of AUC

The implementation of AUC notably affected the PCI rate in actual clinical use. Reported trends indicated a decline in the inappropriate PCI rate [31] for elective/non-acute PCIs or in SIHD patients [18,57,71] since the AUC were released. Between July 2009 and December 2014, the proportion of inappropriate non-acute PCIs decreased from 26.2% to 13.3% (*p* < 0.001) [71]. The decline in the proportion of inappropriate PCIs was reasonable due to the implementation of the AUC, but the rising proportion [71] of the urgency of PCI revealed that the reductions in inappropriate PCI use may reflect changes in documentation or even intentional upcoding, particularly of subjective data elements such as symptom severity [71]. Hannan et al. [31] reported that the percentage of inappropriate PCIs dropped from 18.2% in 2010 to 10.6% in 2014 in New York, which might reflect a deliberate attempt to fit to the new policy. Medicaid reimbursements have been linked to AUC adherence in New York State since 2011 [31], after which comparable increases in coding for AMI and corresponding decreases in coding for UA and non-ACS indications were observed [57]. Such findings led to the concern that AUC may have incentivized some physicians and hospitals to upcode stable angina to UA to conform to the AUC. Future AUC should be modified and upgraded to deal with this concerning trend. Governments should also pay more attention to the artificial, non-medical purpose change in actual clinical use and build a supervision system to monitor it.

## 5. Conclusions

In conclusion, we observed a wide range of inappropriate/rarely appropriate PCI rates in existing studies. The pooled inappropriate/rarely appropriate PCI rate was 4.3% among ACS patients or acute PCIs and 8.9% among non-acute/elective PCIs or non-ACS/SIHD patients. The pooled inappropriate/rarely appropriate overall PCI rate was 6.1%. No difference was found in the inappropriate PCI rate when studies were stratified by location, the country’s degree of development, or the presence of CTO. Inappropriate PCI did not have a significant impact on patients’ outcomes but did lower symptom relief, thereby limiting the clinical utility of AUC. Local AUC are recommended in appropriateness evaluations. Cardiologists could conveniently evaluate the appropriateness of PCI in aggregated patients and provide feedback to providers regarding how individual care decisions match those from a larger population by using AUC.

## Figures and Tables

**Table 1:** Characteristics of the included studies.

Author	Year	Location of Study	Survey Date	AUC	Included PCI Type	Sample Size
Aijaz [22]	2016	Pakistan	Unknown, lasted for 3 years	America 2012 AUC	All PCIs and acute and non-acute PCIs	3328
Barbash [23]	2012	Washington, US	Before and after the publication of the AUC	America 2009 AUC	Not distinguished	2026
Bradley [18]	2015	Washington, US	1 January 2010–31 December 2013	America 2012 AUC	All PCIs and acute and non-acute PCIs	986
Bradley [24]	2015	US and Canada	1999–2004	America 2012 AUC	Non-acute PCI	47,405
Brener [25]	2009	New York, US	Unknown	America 2009 AUC	Non-acute PCI	2134
Chan [26]	2013	US	1 July 2009–31 March 2011	America 2009 AUC	Non-acute PCI	221,254
Chan [27]	2020	US	1 April 2018–30 June 2019	America 2009 AUC	Non-acute PCI	213,753
Chen [28]	2016	Boston, USA	1 June 2013–30 April 2014	America 2012 AUC	Non-acute PCI	277
Gershlick [29]	2012	Leicester, UK	Unknown	America 2009 AUC	Non-acute PCI	200
Gurm [30]	2018	US	1 July 2014–30 June 2015	America 2012 AUC	All PCIs and non-acute PCI	484,722
Hannan [31]	2017	New York, US	2010–2014	America 2012 AUC	Non-acute PCI	67,390
Hess [32]	2019	US	1 November 2013–31 October 2015	America 2012 AUC	Non-acute PCI	2622
Iwasaki [33]	2016	Japan	31 May 2013–30 May 2015	America 2012 AUC	Non-acute PCI	291
Jeon [10]	2017	South Korea	1 January 2014–31 December 2014	Korea KP3 classes	Not distinguished	44,967
Jeptha [17]	2019	US	1 January 2010–31 December 2011	America 2012 AUC	All PCIs and acute PCI	1,123,628
Kawakami [34]	2022	Japan	January 2014–December 2019	America 2017 AUC	Non-acute PCI	5062
Kiselev [35]	2014	Russia	2010–2011	America 2012 AUC	Acute PCI	7244
Ko [36]	2012	Ontario, Canada	1 April 2006–31 March 2007	America 2009 AUC	Non-acute PCI	654
Kohsaka [37]	2014	Tokyo, Japan	September 2008–March 2013	Japan 2007 AUC	Non-acute PCI	2077
Leonardi [38]	2017	Italy	January 2014–May 2016	America 2012 AUC	Not distinguished	401
Min [39]	2013	St. Louis, US	June 2010–January 2011	America 2009 AUC	Not distinguished	422
Mulukutla [40]	2013	Pittsburgh, US	October 2011–April 2012	America 2009 AUC	Non-acute PCI	442
Patil [41]	2017	Maharashtra, India	January 2009–December 2014	America 2012 AUC	Non-acute PCI	894
Puri [42]	2016	Chicago, US	2012–2013	America 2012 AUC	Not distinguished	2054
Ranganayakulu [43]	2014	Tirupati, India	1 August 2013–30 April 2014	America 2012 AUC	All PCIs and acute and non-acute PCIs	978
Sanchez [44]	2014	Pittsburgh, US	May 2012–July 2013	America 2009 AUC	Not distinguished	55
Sastroasmoro [21]	2021	Indonesia	2017–2018	America 2017 AUC	All PCIs and acute and non-acute PCIs	405
Sattur [45]	2012	Sayre, US	Unknown	America 2009 AUC	Acute PCI	112
Saxon [46]	2020	US	21 January 2014–22 July 2015	America 2012 AUC	Non-acute PCI	769
Seixas [47]	2017	São Paulo, Brazil	1 January 2012–31 December 2013	America 2012 AUC	Not distinguished	1070
Senguttuvan [48]	2014	New York, US	January 2010–January 2011	America 2009 AUC	Non-acute PCI	2111
Sood [49]	2016	Karnataka, India	1 October 2014–31 December 2014	America 2012 AUC	Not distinguished	300
Strom [50]	2020	Massachusetts, US	18 December 2016–19 January 2018	America 2017 AUC	Non-acute PCI	121
Takahiro [19]	2014	Japan, Tokyo	September 2008–March 2013	America 2012 AUC	All PCIs and acute PCI	10,050
Waksman [51]	2013	Washington, US	July 2009–July 2011	America 2009 AUC	Non-acute PCI	3152
Wijeysundera [52]	2014	Toronto, Canada	November 2008–December 2009	America 2009 AUC	Non-acute PCI	217
Zheng [53]	2020	Beijing, China	August 2016–August 2017	China 2016 AUC	Non-acute PCI	3677

**Table 2:** The difference in the inappropriate PCI rate between acute and non-acute PCIs in studies presenting both urgent statuses.

Author	Year	Inappropriate Acute PCI	Acute PCI Observed	Inappropriate Acute PCI Rate	Inappropriate Non-Acute PCI	NON-Acute PCI Observed	Inappropriate Non-Acute PCI Rate	*p* Value
Chan [16]	2011	3893	350,469	1.11%	16,838	144,737	11.63%	<0.001
Jeptha [17]	2019	5860	935,845	0.63%	43,251	179,529	24.09%	<0.001
Bradley [18]	2015	224	38,909	0.58%	1785	8496	21.01%	<0.001
Takahiro [19]	2014	146	5100	2.86%	745	2429	30.67%	<0.001
Bradley [23]	2012	84	8010	1.05%	319	1914	16.67%	<0.001
Aijaz [22]	2016	117	2694	4.34%	18	634	2.84%	0.084
Ranganayakulu [43]	2014	61	792	7.70%	10	186	5.38%	0.268
Sastroasmoro [21]	2021	0	214	0.00%	5	191	2.62%	0.017

## Data Availability

All data and materials used in this research are freely available in electronic databases. References have been provided.

## Supplementary Materials

The following supporting information can be downloaded at: https://www.mdpi.com/article/10.3390/jcdd10030093/s1, Item S1: Search strategy; Figure S1: Risk of bias graph of the included studies; Figure S2: Risk of bias graph of the included studies; Figure S3 Funnel plot of the inappropriate PCI rate of non-acute PCIs; Figure S4: Funnel plot of the inappropriate PCI rate of all PCIs; Figure S5: Graphical summary.
